# A review on the genus *Kadsura*: ethnobotany, pharmacology, and molecular pharmacognosy

**DOI:** 10.3389/fphar.2026.1768643

**Published:** 2026-03-11

**Authors:** Niaojiao Xu, Xianjing Li, Yue Zhang, Lu Qu, Haitao Li, Yunqiang Wang, Meifang Song, Baozhong Duan, Zhonglian Zhang

**Affiliations:** 1 College of Pharmacy, Dali University, Dali, China; 2 Yunnan Key Laboratory of Southern Medicine Utilization, Yunnan Branch of Institute of Medicinal Plant Development, Chinese Academy of Medical Sciences, Peking Union Medical College, Jinghong, China

**Keywords:** ethnobotany, *Kadsura*, molecular pharmacognosy, pharmacology, propagation

## Abstract

*Kadsura* Kaempf. ex Juss., belonging to the Schisandraceae family, is a key ethnomedicinal resource in traditional Asian medicine, valued for both dietary and therapeutic roles. Traditionally used to boost blood circulation, relieve pain, and dispel wind-cold-damp pathogens, its metabolites have been validated by modern pharmacology to exhibit potent anti-rheumatoid arthritis, hepatoprotective, antioxidant, and anti-inflammatory properties, supporting clinical applications for rheumatic and hepatic conditions. Literature was retrieved from major databases (Google Scholar, Web of Science, PubMed, ScienceDirect, Baidu Scholar, CNKI, etc.), monographs, and dissertations using “*Kadsura*” as the core keyword. Species identities were verified via Plants of the World Online (http://www.plantsoftheworldonline.org), and data on ethnobotany, pharmacology, and molecular pharmacognosy were rigorously screened and synthesized. Our analysis reveals that while *Kadsura* species share core medicinal attributes, the scientific basis for their species-specific traditional therapeutic effects remains unclear. Furthermore, the mechanisms of some pharmacological activities are not fully elucidated, systematic safety evaluations are insufficient, and molecular pharmacognosy research is limited to preliminary transcriptomic and metabolomic screening and expression profiling. Meanwhile, systematic reviews on these aspects remain lacking. To address this gap, this review summarizes the ethnobotany, pharmacology, and molecular pharmacognosy of the genus to date, laying a theoretical foundation for further development and utilization (*Kadsura ananosma* Kerr, a synonym of *Kadsura coccinea* (Lem.) A.C.Sm.; *Kadsura polysperma* Y.C.Yang and *Kadsura interior* A.C.Sm., a synonym of *Kadsura heteroclita* (Roxb.) Craib).

## 1 Introduction

The genus *Kadsura*, belonging to the Schisandraceae family, comprises 29 species predominantly distributed across eastern and southeastern Asia (Guo and Tang, 2021), including China, South Korea, Japan, the Philippines, and Thailand (Sritalahareuthai et al., 2020a; Tai et al., 2022). As a key member of the Schisandraceae family, this plant genus is widely distributed and possesses a long history of medicinal application. Throughout the history of traditional medicine, plants of the genus *Kadsura* have attracted considerable attention for their remarkable therapeutic properties. Modern pharmacological studies have demonstrated that these plants exhibit strong anti-HIV, antitumor, antioxidant, anti-platelet aggregation, and neuroprotective effects (Liu et al., 2014). Meanwhile, worldwide, the fruits of many *Kadsura* species also have excellent edible value.

Plants of the *Kadsura* genus are widely used as traditional folk medicinal materials with various traditional medicinal activities and have great development potential. However, many species of this genus have not yet been studied. Summarizing their medicinal experience is of great significance for the discovery of new chemical substances and the development of new drugs derived from plants of the *Kadsura* genus. Therefore, it is necessary to summarize current research progress on time and, in combination with traditional medication experience, promptly identify weak research directions and strengthen targeted research in this field, providing a basis for the in-depth development of the clinical application value of *Kadsura* plants. Furthermore, with increasing global demand for *Kadsura* plants and rising awareness of ecological protection, research into their introduction, cultivation, and propagation has become crucial. In addition, Molecular pharmacognosy is an interdisciplinary subject derived from molecular biology, specializing in the identification, quality evaluation, and characterization of the molecular constituents of Chinese medicinal materials. In-depth research on the molecular pharmacognosy of the *Kadsura* plants will provide more scientific and effective technical support for the further development of their medicinal value, and effectively promote the high-quality and sustainable development of the *Kadsura* industry.

**FIGURE 1:**
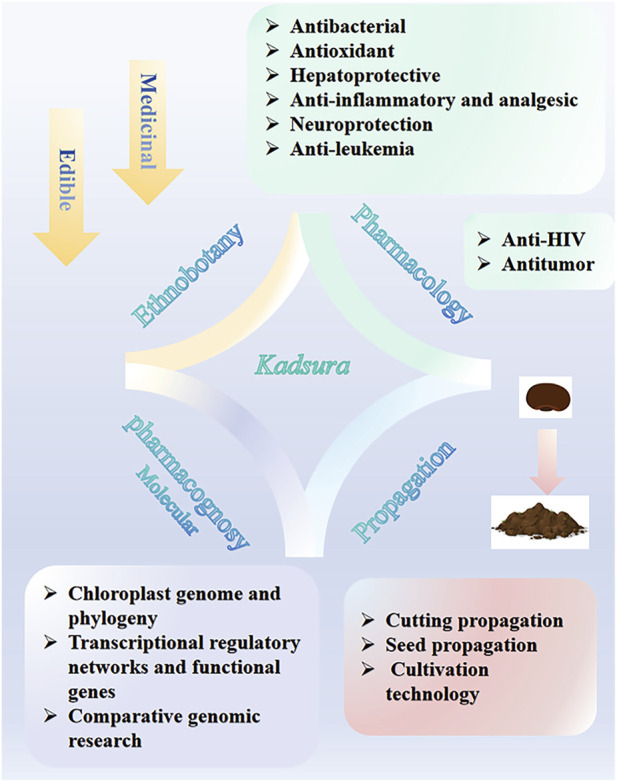
Current research progress of genus *Kadsura*.

Therefore, this article reviews the progress in its edible characteristics, ethnobotany, and pharmacological research (Figure 1), and proposes further research strategies to provide theoretical support for its modern clinical application in traditional medicine and to lay a foundation for its development in medicine and food. In addition, this article reviews the research progress on *Kadsura* plants in reproduction and molecular pharmacognosy, providing a basis for analyzing the biosynthetic pathways of their active metabolites and for studying sustainable utilization.

## 2 Ethnobotany

### 2.1 Edible properties

The fruits of multiple species of the genus *Kadsura* can be eaten. In Thailand, the fruits of *K. coccinea* (Lem.) A.C.Sm. and *Kadsura heteroclita* (Roxb.) Residents widely consume Craib. Researchers conducted a systematic assessment of the nutritional value of different parts of these fruits (including exocarp, mesocarp, seeds, and pits) (Sritalahareuthai et al., 2020a), verifying their safety for consumption and their nutritional value. In China, the fruits of plants such as *K. coccinea*, *Kadsura longipedunculata* Finet and Gagnep., and *Kadsura oblongifolia* Merr. are also widely consumed as fruits (Idrees et al., 2022; Long et al., 2022; Dong et al., 2023). Among them, the research on *K. coccinea* is the most extensive. Its fruit has a variety of skin colors (including green, pink, bright red, purplish red, red-black, etc.), is large, non-toxic, and has a sweet-and-sour, juicy taste. Mature *K. coccinea* seeds are rich in fatty acids, amino acids, vitamins, and minerals, and have healthcare functions such as antioxidation, anti-fatigue, lowering blood lipids and blood pressure, etc., which could meet the material basis conditions for modern human health preservation and balanced nutrition (Li et al., 2025; Liu, 2024; Wang, 2022; Zhang et al., 2022; Zhu et al., 2022). Excitingly, product development for *K. coccinea* has expanded into the cosmetics industry. Its fruit extract has been included in the “Directory of Names of Used Cosmetic Raw Materials” by the national food and drug regulatory authorities. Currently, five cosmetic products have been developed from the *K. coccinea* metabolite. Meanwhile, relevant research indicates that its leaf extract also has potential for development as a cosmetic raw material (Ying et al., 2024). Due to the continuous increase in market demand and the growing scarcity of wild resources, southern provinces such as Hunan, Guizhou, and Yunnan in China have already achieved considerable areas of artificial cultivation.

In conclusion, the fruits of many *Kadsura* species are consumed by people and have definite medicinal value, such as those of *K. coccinea*, *K. heteroclita*, and *K. oblongifolia*. Among them, *K. coccinea* is currently the most widely used, extensively studied, and largest cultivated species.

### 2.2 Medicinal properties

The majority of plants in the *Kadsura* genus have shown considerable therapeutic efficacy, and their traditional medicinal use dates back many years (Table 1). In traditional medicine, the roots, stems, and leaves of these plants are frequently used as botanical drugs, mainly to promote blood circulation, reduce pain, and dispel wind-dampness (Song, 1988; Xu et al., 2008; Zhang et al., 2021; Xiao et al., 2010). Additionally, the fruits of the *Kadsura* species are widely used for health preservation and possess noteworthy therapeutic properties. Additionally, the fruits of the *Kadsura* species exhibit notable medicinal properties (Guo, 2016; Huang et al., 2021) and are widely utilized in health preservation. For example, *K. coccinea* is valued not only for its medicinal properties but also widely consumed as a fruit in Thailand (Sritalahareuthai et al. (2020a)). Their unique pharmacological properties and nutritional profiles have garnered increasing scientific attention and recognition.

**TABLE 1:** Folk applications of *Kadsura* plants.

Species names	Ethnic	Common names	Traditional efficacy and uses	Parts
*K. coccinea*	Miao, Zhuang, Yao, Tujia, Dong, Dai, Hani, Li, and Da	Len Fantuan, Chou Fantuan, Ru Dishexiang, Shi Bazheng, Hong Zuan	Promotes qi circulation, relieves pain, disperses stasis, reduces swelling, relaxes tendons, and activates collaterals. Treats rheumatoid arthritis, back and leg pain, chronic gastritis, gastric and duodenal ulcers, dysmenorrhea, postpartum abdominal pain, hernia pain, injuries from falls, etc.	Root, bark, stem, leaf
*K. heteroclita*	Dai, Hani, Jinuo, Mulao, Miao, Wa, Zhuang, Tujia	Chui Fengsan, Zhu Naiguo, Len Fantuan, Tong Xuexiang, Di Xuexiang, Feng Qing Jixueteng, Dian Jixueteng	Regulates menstruation, promotes blood circulation, relieves pain, expels wind, and alleviates pain. Treats enteritis, rheumatoid arthritis, gastric and duodenal ulcers, injuries, fractures, external bleeding, lumbar strain, rheumatic bone pain, sciatica, dysmenorrhea, postpartum abdominal pain, colds, bronchitis, neurasthenia, red and white dysentery, etc	Root, stem, fruit
*K. longipedunculata*	Dai, Zang, Yi, Miao, Zhuang, Yao, Buyi, Dong, Gelao, Da, Tujia	Hong Muxiang, Zi Jingteng, Zi Jingpi, Xiao Xueteng, Man Shanxiang, Xiang Su	Activates blood, regulates qi, expels wind, promotes joint health, reduces swelling, and relieves pain. Treats stomach pain, abdominal pain, rheumatic pain, dysmenorrhea, irregular menstruation, postpartum abdominal pain, throat swelling, hemorrhoids, undiagnosed swelling, injuries, cuts, cough, poor appetite, night sweats, neurasthenia, etc.	Root, leaf, fruit
*Kadsura interior* A.C.Sm.	Dai, Miao	Feng Qiangjixueteng, Dian Jixueteng	Clears heat and detoxifies; regulates menstruation; relieves pain; nourishes blood; stops bleeding; and alleviates dysentery. Primarily treats abdominal pain, diarrhea, red and white dysentery, postpartum weakness, etc.	Stem
*Kadsura angustifolia* A.C.Sm.	Yi	​	Promotes blood circulation, removes stasis, regulates qi, and relieves pain. Treats irregular menstruation, rheumatism, fractures, external bleeding, etc.	Whole plant
*Kadsura japonica* (L.) Dunal	Da	Zuan Gufeng, Wen Niuteng, Feng Teng	Treats lung deficiency cough, chronic diarrhea, dysentery, abdominal distention, reversed qi flow, boils, and abscesses.	Root, leaf, seed
*K. oblongifolia*	Miao, Yao	Chui Fengsan, Xiao Hongzuan	Treats kidney deficiency and impotence, rheumatic pain, dysmenorrhea, injuries, fractures, enteritis, stomach pain, colds, etc.	Root, stem
*Kadsura renchangiana* S.F.Lan	Yao	Tie Zuan	Expels wind and dampness, reduces swelling, and relieves pain. Treats injuries, swelling, rheumatic bone pain, etc.	Stem
*Kadsura ananosma* Kerr	​	Xiao Xueteng, Da Hongpao	Dispelling wind and dampness, promoting tendon-muscle relaxation, consolidating astringency, replenishing qi and body fluids, tonifying kidneys, and tranquilizing the mind. Treats chronic cough, asthma, nocturnal emissions, palpitations, insomnia, injuries, rheumatic pain, fractures, irregular menstruation, external bleeding, etc.	Root, stem, leaf, fruit

Combining traditional folk medicinal knowledge, people have already conducted corresponding pharmacological activity studies on some species. *K. heteroclita*, found in regions such as China, Sikkim, Bangladesh, Vietnam, Laos, Myanmar, Thailand, India, and Sri Lanka (Editorial Committee Of Flora Reipublicae Popularis Sinicae, 2004), demonstrates significant pharmacological benefits, including anti-inflammatory, anticancer, and hepatoprotective effects (Wang et al., 2021; Yu et al., 2019a; Yu et al., 2019b; Deng and Zhang, 2016). Sritalahareuthai et al. (2020b) reported that the phenolic profiles of this species exhibit potent antioxidant and inhibitory activities. The vine stems of *K. longipedunculata*, traditionally known as “Hongmuxiang” in China, are widely used to treat inflammatory and infectious conditions. In Germany, they are appreciated for their diverse pharmacological activities, including antimicrobial, trypanocidal, anti-inflammatory, and cytotoxic effects (Mulyaningsih et al., 2010). Furthermore, revealed that its lignans play a significant role in modulating the GABA A receptor. Similarly, the lignans in *K. interior* have demonstrated inhibition of ADP-induced platelet aggregation (Liu et al., 2018b).

The species involved in this article exhibit both similarities and distinct differences in their traditional applications. For example, most of the *Kadsura* species listed in Table 1 have the effects of promoting qi circulation and relieving pain, activating blood circulation and removing blood stasis, dispelling wind and dampness. They are mainly used to treat symptoms such as injuries from falls and blows, rheumatic pain, and menstrual disorders. In addition, some species have unique medicinal properties. For instance, *K. interior* possesses the unique effects of clearing heat and detoxifying, as well as replenishing and stopping bleeding among the species of the *Kadsura* genus. *K. japonica* has the effect of treating lung deficiency cough, *K. oblongifolia* has the effect of treating kidney deficiency impotence, and *K. ananosma* has the effects of astringency, tonifying qi, and nourishing the kidney. The above fully demonstrates the chemical diversity of the *Kadsura* species and the unique chemical properties of each species.

These species are integrated into various regional folk medicine practices, although their specific applications and therapeutic effects vary. Preliminary analyses suggest that these discrepancies may arise from differences in chemical compositions, growing environments, harvesting seasons, and the theoretical and practical frameworks of traditional medicine, which contribute to their unique medicinal profiles. Ongoing research continues to reveal the diverse ethnic uses and potential applications of the genus *Kadsura*, providing new insights and resources for modern drug development.

### 2.3 Cultivation

#### 2.3.1 Propagation

Scholarly investigations into propagation techniques for the genus *Kadsura*, including cutting propagation, seed germination, and tissue culture, have largely been confined to a narrow subset of species, leading to significant disparities in the depth and breadth of research across taxa. With respect to cutting propagation, *K. ananosma* exhibits optimal growth and survival rates of up to 86% when cultured in a substrate consisting of 35% forest topsoil blended with 65% perlite (Li and Liu, 2014). For *K. longipedunculata*, stem cuttings measuring 9∼10 cm in length yield survival rates exceeding 60%, with thicker stem segments positively correlating to improved survival outcomes (Lin et al., 2015). In contrast, *K. coccinea* has been the subject of the most intensive research on cutting propagation. Suitable substrates include humus-rich soil and slightly acidic loam (Wu, 2018; Zhang, 2018), while pre-treatment of stem segments with 100 mg/L naphthalene acetic acid (NAA) significantly enhances rooting efficiency (Huang et al., 2018). Furthermore, when cuttings are grown in a plastic greenhouse with 70% light transmittance, survival rates can exceed 95%, underscoring the critical role of environmental conditions in cutting propagation success (Lin and Zhou, 2021). Moreover, field cultivation with a planting density of 1.5 m × 2 m and a single-layer trellis system results in a survival rate of 68.3% (Yin et al., 2017).

Turning to seed propagation, most research on *Kadsura* species has focused exclusively on *K. coccinea*. Studies demonstrate that seed treatment with either a mixed solution of 0.5% CaCl_2_ and 50 μg/mL gibberellic acid (GA) (Zhao and Zhang, 2011) or a 30 mg/L GA solution alone (Li and Liang, 2018) can boost germination rates to over 90%. Soaking seeds in optimal concentrations of plant growth regulators not only breaks seed dormancy and stimulates germination but also enhances seedling vigor; notably, the combined application of 200 mg/L 6-benzyladenine (6-BA) and 100 mg/L salicylic acid (SA) further improves germination efficacy (Jiang, 2023). Additional effective dormancy-breaking treatments include: soaking seeds in concentrated sulfuric acid for 30 s (83.77% germination rate), a 60 °C hot water bath (68.53% germination rate), cryogenic treatment in liquid nitrogen for 20 min (82.47% germination rate), and immersion in 100 mg/L GA solution (89.96% germination rate) (He, 2022). Furthermore, 60 days of cold stratification followed by 30 days of incubation results in a germination rate of 78.18% (He, 2022).

In comparison, research on tissue culture techniques for *Kadsura* species remains relatively scarce, with current studies limited to *K. coccinea*. Lu et al. (2022) identified optimal media for *K. coccinea* shoot tip culture: 1/2 MS medium supplemented with 2.0 mg/L 6-BA and 0.2 mg/L indole-3-acetic acid (IAA) for primary culture, and 1/2 MS medium with 3.0 mg/L 6-BA and 0.1 mg/L 3-indolebutyric acid (3-IBA) for subculture. Deng et al. (2024) explored seed-based tissue culture for *K. coccinea*, developing specialized media for proliferation and rooting that achieved a 100% rooting rate and robust seedling growth. They also established optimal acclimatization durations, transplant substrates, shading protocols, and cultivation cycles for tissue-cultured plantlets.

#### 2.3.2 Cultivation technology

Research on cultivation techniques for medicinal plants of the *Kadsura* genus has focused on two species: *K. coccinea* and *K. longipedunculata*. Among these, *K. coccinea* has been the most extensively studied. Two primary planting seasons have been identified for *K. coccinea*: spring (late February to early March) and autumn (late October to early November) (Chen, 2015; Tao et al., 2002). This species demonstrates shade tolerance, strong stress resistance, and adaptability to a wide temperature range (both low and high) (Qi et al., 2014; Yin et al., 2017; Liang et al., 2017). Optimal growth conditions include slightly acidic sandy soils or loose, fertile loam, paired with a light intensity of 30%–50% (Wu, 2012).

Furthermore, soil phosphorus content, cation exchange capacity, and pH are recognized as critical yield determinants (Liang and Li, 2019). Thus, soil selection, targeted fertilization, and pH adjustment are essential for successful cultivation and optimal yield. For *K. longipedunculata*, seeds should be collected between September and October upon full fruit maturation and sown via row-sowing from March to April. And we recommend transplanting in the autumn of the same year or in the spring of the following year (Wang, 2013).

#### 2.3.3 The impact of environmental factors

Research shows that environmental factors, such as light, water, and altitude, significantly influence the growth of *Kadsura* plants. For example, Zhong et al. (2009) suggested that moderate shading is beneficial for *K. japonica* growth, as it enhances the activity of various enzymes under these conditions. In the case of *K. coccinea*, light plays a particularly critical role. The optimal light intensity for its growth ranges between 30% and 50% (Qi et al., 2014; Zhao et al., 2021), and it is advisable to cultivate this species in areas with ample water sources to ensure robust development (Yang et al., 2020). Additionally, *K. ananosma* can grow normally in tropical regions at an altitude of 1,300 m (Yang et al., 2016). In Xishuangbanna, it is best to select plots at an altitude of about 1,000 m for optimal growth (Yuan et al., 2019). Wu et al. (2022) noted that *K. longipedunculata* is more suited to growing on shady or semi-shady slopes with high humidity. It thrives in loose, well-aerated sandy loam, gravelly soil, or yellow clay soil at altitudes below 800 m.

In summary, researchers have conducted studies on cuttings propagation techniques for various species of the genus *Kadsura*, including *K. ananosma*, *K. longipedunculata*, and *K. coccinea*. The survival rate of cuttings depends not only on the quality of the cutting material itself but also on factors such as the selection of rooting substrates, the application of rooting hormones, and field management practices. Significant progress has been made in the cultivation research of multiple *Kadsura* species: standardized propagation protocols for cuttings and seeds have been established for species such as *K. coccinea*, achieving cuttings survival rates exceeding 95% and seed germination rates over 90%. Additionally, cultivation techniques have identified key conditions, including planting seasons, soil types, and light requirements, while environmental adaptability studies provide a scientific basis for precise site selection, thereby offering solid support for large-scale cultivation.

While these advancements lay a solid foundation for practical application, notable limitations persist in the current research landscape: uneven species coverage remains a critical issue, with in-depth studies concentrated on *K. coccinea* and research gaps across most other species; tissue culture technology is restricted to *K. coccinea* and remains underdeveloped overall; environmental factor analysis primarily focuses on single variables, lacking investigations into multi-factor interactions; and comprehensive comparative studies on alternative propagation methods and cross-regional cultivation techniques are insufficient. These gaps collectively hinder the full development, utilization, and technological dissemination of the genus as a whole.

## 3 Pharmacology

### 3.1 Hepatoprotective effects

Metabolites derived from *Kadsura* species exert hepatoprotective effects by targeting three core signaling networks, with distinct species-specific variations in pathway prioritization and regulatory mechanisms. First, the regulation of oxidative stress through activation of the Nrf2/ARE signaling axis represents a conserved hepatoprotective mechanism across multiple *Kadsura* species. For instance, the ethanol extract of *K. coccinea* has been shown to robustly upregulate the expression of superoxide dismutase (SOD) and glutathione peroxidase (GPX-2), while concurrently suppressing the levels of malondialdehyde (MDA) and myeloperoxidase (MPO) in the livers of mice with carbon tetrachloride (CCl_4_)-induced injury (Yu et al., 2021). Mechanistically, this protective effect is likely mediated by binankadsurin A that directly interacts with Kelch-like ECH-associated protein 1 (Keap1) to facilitate the nuclear translocation of Nrf2. However, direct binding assays are required to validate this proposed mechanism experimentally.

Second, the inhibition of inflammatory responses through the NF-κB and MAPK signaling pathways exhibits notable species-specific divergence. Specifically, *K. coccinea* suppresses the secretion of pro-inflammatory cytokines TNF-α and IL-6 by reducing the phosphorylation of c-Jun N-terminal kinase (JNK) and p38 mitogen-activated protein kinase (p38) (Wu et al., 2024; Li et al., 2010; Liu et al., 2015; Qu et al., 2003; Zhu, 2007; Liu et al., 2018c; Liu et al., 2018d); in contrast, *K. heteroclita* harbors hepatoprotective metabolites, including kadsurarin, meso-dihydroguaiaretic acid, kadsuphilol C, and coumarinlignan (Wang et al., 2022; Su et al., 2015), these may also function through the same mechanism. Third, the attenuation of hepatic fibrosis through HSC inactivation is a key hepatoprotective feature observed in several *Kadsura* species. For example, extracts of *K. coccinea* inhibit the proliferation of HSC-T6 cells and induce apoptosis through lignan metabolites, such as acetylpigomisin R (Ban et al., 2009). Similarly, certain metabolites in *K. longipedunculata*, including kadsuraols C, schiarisanrin, micrandilactone I, B22,23-di-epi-micrandilactone J, and longipedlignans F, also play significant roles (Shao et al., 2020; Wang et al., 2017; Wang et al., 2018; Liu et al., 2016; Liu et al., 2018a). Collectively, these findings support the overarching hypothesis that the structural diversity of *Kadsura* metabolites determines their pathway selectivity: lignans preferentially target oxidative stress and inflammatory responses, whereas terpenoid-lignan hybrids exhibit superior efficacy in inhibiting fibrosis-related HSC activation.

The species-specific profiles of bioactive metabolites further highlight the significant therapeutic potential of the *Kadsura* genus. For instance, *K. coccinea* primarily relies on acetylated lignans (acetylpigomisin R, binankadsurin A) to protect hepatocytes against tert-butyl hydroperoxide-induced damage, with acetylation modifications significantly enhancing their bioactivity (Ban et al., 2009). In contrast, *K. heteroclita* exerts its hepatoprotective effects through the combined action of metabolites (meso-dihydroguaiaretic acid, kadsurarin), which synergistically modulate oxidative stress and inflammatory signaling pathways (Su et al., 2015). On the other hand, the terpenoid-lignan hybrid metabolites of *K. longipedunculata* (micrandilactone I, longipedlignans F) demonstrate superior anti-fibrotic activity when compared to single-class metabolites (Liu et al., 2018a). These species-specific differences underscore the critical need for targeted research strategies to optimize the selection of lead metabolites for the treatment of distinct liver disease subtypes.

Despite the promising preclinical findings, the translation of *Kadsura*-derived agents into clinical practice is hindered by several critical bottlenecks. First, most lignans exhibit low oral bioavailability, primarily due to their poor water solubility and susceptibility to first-pass metabolism. To address this issue, structural modifications or formulation strategies, such as nanoliposome encapsulation, may be employed to improve bioavailability and delivery efficiency. Second, significant gaps in standardization persist across *Kadsura* extract preparations, as variable extraction protocols yield inconsistent metabolite profiles. Therefore, the development of marker-based quality control standards. For example, using kadsurarin as a marker metabolite for *K. heteroclita* extract is crucial to ensure the reproducibility of preclinical and clinical studies.

Most importantly, to date, no large-scale clinical trials have been conducted to validate the safety and efficacy of *Kadsura*-derived metabolites for the treatment of liver diseases. Accordingly, future research should prioritize the initiation of phase I/II clinical trials in patients with non-alcoholic steatohepatitis (NASH) or drug-induced liver injury, with primary endpoints including changes in liver enzyme levels and improvements in fibrosis staging. By systematically addressing these translational challenges and rigorously testing the proposed mechanistic hypotheses, species of the *Kadsura* genus hold significant potential to expand the current therapeutic arsenal for the management of liver disorders.

### 3.2 Antitumor effects

Multiple studies have confirmed that extracts from *Kadsura* species exert robust anticancer effects. *K. heteroclita* stands as one of the most thoroughly investigated species within the genus, with its lignan and triterpenoid metabolites exhibiting robust cytotoxicity against a broad panel of cancer cell lines. For instance, the lignan (+)-1-hydroxy-2,6-bis-epi-pinoresinol and triterpenoid xuetonglactone F from *K. heteroclita* significantly inhibit the viability of BGC-823 gastric cancer cells and HeLa cervical cancer cells, with notable selectivity compared to normal somatic cells (Shehla et al., 2019; 2022). Furthermore, crude triterpenoid extracts from *K. heteroclita* have been shown to induce caspase-dependent apoptosis in multiple human cancer cell lines, including OVCAR, HT-29, Hep-G2, and A549, with IC_50_ values ranging from 16.2 to 36.4 µM (Xu, 2006; Minh et al., 2014). Notably, among its purified triterpenoid metabolites, longipedlactones A and F exhibit exceptionally potent cytotoxicity against Hep-G2 and Bel-7402 hepatocellular carcinoma cell lines, highlighting their potential as lead metabolites for subsequent drug optimization and development (Xu et al., 2010).

Beyond *K. heteroclita*, other *Kadsura* species contribute a structurally diverse repertoire of antitumor metabolites, with activities spanning both solid tumors and hematological malignancies. For example, volatile oil metabolites isolated from *K. longipedunculata*, such as camphene and borneol, exhibit moderate cytotoxicity against Hep-G2 hepatocellular carcinoma, MIA-Paca-2 pancreatic cancer, and SW-480 colorectal cancer cells, with IC_50_ values of 133.53, 136.96, and 136.62 mg/mL, respectively (Mulyaningsih et al., 2010). Moreover, the triterpenoid-rich fraction of *K. longipedunculata*, including metabolites like kadlongilactones A–F and longipedlactones A–C, F, H, demonstrates potent cytotoxicity against K562 chronic myeloid leukemia cells (IC_50_: 0.84–11.38 µM) and solid tumor cell lines such as A549 lung cancer and HT-29 colorectal cancer (IC_50_: 0.49–3.61 µM) (Pu et al., 2005; 2007; Song et al., 2021). Furthermore, penochrochlactone C, a unique triterpenoid isolated from *K. longipedunculata*, inhibits the proliferation of HeLa cervical cancer cells with an IC_50_ of 9.70 µM (Song et al., 2021). Similarly, taiwankadsurin B, a neolignan from *Kadsura philippinensis* Elmer, exhibits dose-dependent cytotoxicity against a range of human tumor cell lines, further expanding the structural diversity of antitumor agents within the genus (Shen et al., 2005).

In addition to their direct cytotoxic effects against established cancer cells, *Kadsura* species also hold significant chemopreventive potential by targeting key tumor-promoting pathways. For example, fourteen neolignans isolated from various *Kadsura* species have been identified as potent inhibitors of 12-O-tetradecanoylphorbol-13-acetate (TPA)-induced Epstein-Barr virus early antigen (EBV-EA) activation in Raji cells, with neokadsuranin and schisandrin C displaying the highest inhibitory potency (Chen D. et al. 2002). Critically, *in vivo* studies have validated the therapeutic efficacy of *Kadsura* extracts in preclinical animal models. For instance, the chloroform extract of *K. coccinea* significantly suppresses tumor growth and induces apoptosis in a dose-dependent manner in a murine 4T1 breast cancer xenograft model. Mechanistically, this extract exerts its antitumor effects by modulating the MAPK signaling cascade—specifically by upregulating the phosphorylation of ERK, JNK, and p38 MAPK, while concurrently regulating cytokine expression profiles (upregulating pro-inflammatory mediators IL-6, IL-1β, iNOS, and TNF-α; downregulating COX-2 to reshape the tumor microenvironment into an antitumor state (Jin, 2022).

Collectively, these preclinical findings robustly underscore the immense potential of *Kadsura* species as a prolific source of novel anticancer and chemopreventive agents. The structural diversity of their bioactive metabolites, combined with their multi-targeted mechanisms of action, positions these metabolites as promising candidates for further structural optimization, rigorous *in vivo* validation using immunocompetent models and patient-derived xenografts (PDXs), and eventual translation into clinical trials to address critical unmet needs in cancer therapy and chemoprevention.

### 3.3 Antimicrobial effects

To date, three *Kadsura* species have been scientifically validated for their antibacterial properties: *K. coccinea* (Feng et al., 2011; Yuan and Zhong, 2009), *K. longipedunculata* (Li, 2015; Lin et al., 2017; Lin et al., 2019; Liu et al., 2013; Wang et al., 2013; Wu et al., 2012; Lin et al., 2018), and *K. angustifolia* (Song et al., 2021), each exhibiting distinct species-specific antibacterial profiles that are tightly correlated with their unique secondary metabolite compositions.

Specifically, the ethanolic extract of *K. coccinea* fruit peel exhibits selective inhibitory activity against Gram-negative *Salmonella typhi* (Feng et al., 2011) and Gram-positive *Streptococcus* pneumoniae (Yuan and Zhong, 2009), though its inhibitory efficacy against clinically relevant pathogens such as *Bacillus* cereus and methicillin-resistant *Staphylococcus aureus* (MRSA) remains relatively limited. In contrast, *K. longipedunculata* represents the most extensively investigated species within the genus, with its aqueous and ethanolic extracts demonstrating robust broad-spectrum antibacterial activity against both Gram-positive and Gram-negative bacterial pathogens; notably, it exerts potent inhibitory effects against multidrug-resistant *Staphylococcus aureus* (MRSA), a leading cause of nosocomial and community-acquired infections (Li, 2015; Liu et al., 2013; Wang et al., 2013). Meanwhile, *K. angustifolia*, a relatively understudied yet promising species, exhibits moderate antibacterial activity against a panel of clinically significant pathogens, including methicillin-sensitive *Staphylococcus aureus* (MSSA), *Bacillus subtilis*, *Escherichia coli*, and *Pseudomonas aeruginosa* (Song et al., 2021).

While these findings demonstrate that *Kadsura* species possess species-specific antibacterial properties, highlighting their differentiated therapeutic potential, current studies primarily rely on *in vitro* experiments. Such approaches neither replicate host environments nor assess drug resistance evolution, potentially leading to biased antimicrobial efficacy. Furthermore, evaluations of metabolic stability and *in vivo* distribution remain insufficient. Therefore, integrating *in vivo* experiments with clinical studies is essential to validate their therapeutic potential and safety comprehensively.

### 3.4 Antioxidant effects

Extensive preclinical investigations have systematically validated the robust antioxidative activity of several key *Kadsura* species. For instance, the lignans from *K. coccinea* exhibit multifaceted antioxidative effects: they directly scavenge free radicals including DPPH, ·OH, and superoxide anions; upregulate the activity of endogenous antioxidant enzymes such as SOD; and modulate oxidative stress signaling pathways to suppress melanin synthesis, highlighting potential applications in both disease treatment and cosmetic formulations (Goh et al., 2013; Liu et al., 2018e; Ai and Li, 2005; Li et al., 2020; Tang, 2020; Wang, 2022; Yan et al., 2013; Zuo, 2021). Concurrently, *K. heteroclita* produces a distinct array of antioxidative metabolites, including cytochalasin H, isolariciresinol, and gomisin J, which demonstrate potent reactive oxygen species (ROS)-scavenging capacity and protect against oxidative damage in cellular models of neurodegeneration and liver injury (Shehla et al., 2022; Cao et al., 2019).

Current research on the sources of antioxidants in *Kadsura* remains predominantly descriptive, with most studies relying on simple *in vitro* experiments (free radical scavenging assays) that fail to replicate the complex *in vivo* physiological environment of oxidative stress. Additionally, its underlying molecular mechanisms remain unelucidated.

### 3.5 Anti-inflammatory and analgesic effects

Current research on the anti-inflammatory and analgesic activities of *Kadsura* species primarily focuses on two species: *K. coccinea* and *K. heteroclita* (Li et al., 2006; Yang et al., 2021; Yang et al., 2022; Hu and Chen, 2018; Ji, 2011; Li et al., 2014; Liu and Wang, 1989; Lu, 2021; Shi and Li, 2016; Sun, 2020; Sun et al., 2022; Li, 2023; Yu et al., 2019a; Yu et al., 2019b; Yu, 2017; Zheng et al., 2024). *K. coccinea* exerts broad-spectrum anti-inflammatory and analgesic activity via multi-pathway regulation: in lipopolysaccharide (LPS)-stimulated RAW264.7 macrophages and collagen-induced arthritis (CIA) mouse models, its lignan-rich extracts inhibit the NF-κB and COX-2 signaling cascade to reduce the production of pro-inflammatory cytokines (TNF-α, IL-1β, IL-6), activate the nuclear factor erythroid 2-related factor 2 (Nrf2) antioxidant pathway to mitigate oxidative stress, and directly suppress RA synoviocyte proliferation and osteoclast differentiation, thereby alleviating joint destruction and pain (Lu et al., 2021; Li et al., 2023). In contrast, *K. heteroclita* demonstrates targeted activity particularly relevant to bone-related inflammatory disorders: its unique triterpenoid metabolites (xuetonglactones A/B, xuetongsu) specifically inhibit inducible nitric oxide synthase (iNOS), all COX subtypes, and bone resorption-related proteins (MMP-9, CTSK, TRAP), with IC_50_ values ranging from 3.2 to 12.8 µM against key inflammatory enzymes. This makes it a promising candidate for the treatment of RA and osteoporosis, where excessive bone resorption drives disease progression (Shehla et al., 2022; Cao et al., 2019).

However, current research on *K. longipedunculata* and *K. interior* remains relatively limited; the mechanism of anti-inflammatory action has not been elucidated, most studies rely on *in vitro* assays or rodent models with limited pharmacokinetic data, and no clinical trials have validated human safety or efficacy (Tan et al., 2017; Zhang et al., 1991; Tang et al., 2024; Li et al., 1999b). To facilitate more effective development and utilization, future work should use multi-omics to identify precise molecular targets, validate *in vivo* efficacy in disease-relevant models, optimize formulations to enhance bioavailability, and advance clinical trials to unlock *Kadsura*’s potential as novel anti-inflammatory and analgesic therapies.

### 3.6 Anti-HIV effects

Species of the genus *Kadsura* have emerged as a promising source of natural anti-HIV agents, with triterpenoids and lignans identified as the primary bioactive metabolites exhibiting diverse mechanisms of action against HIV-1 replication (Sun et al., 2011; Gao et al., 2008). Triterpenoid extracts from *K. heteroclita* demonstrate selective cytotoxicity against HL-60 human promyelocytic leukemia cells. This activity is hypothesized to indirectly suppress HIV replication by modulating immune cell populations and reducing viral reservoir formation in infected hosts (Xu, 2006; Xu et al., 2010). Meanwhile, *K. longipedunculata* produces a suite of potent anti-HIV-1 metabolites with distinct modes of action: schisanlactone A, longipedunin A, and lancilactone C inhibit early-stage HIV-1 entry and post-entry viral processing, while the dibenzocyclooctadiene lignan kadlongirin B directly targets the HIV-1 replication machinery, likely interfering with reverse transcriptase activity or viral integration into host chromatin (Sun et al., 2006; Pu et al., 2008). Notably, these metabolites exhibit low cytotoxicity against normal human peripheral blood mononuclear cells (PBMCs), suggesting a favorable therapeutic index for further drug development.

However, the clinical translation of anti-HIV agents derived from the genus *Kadsura* still faces two critical bottlenecks. On the one hand, most existing studies rely on simple *in vitro* assay systems that cannot accurately replicate the complex interactions between HIV and the human immune system, leaving the core molecular mechanisms underlying their anti-HIV activity incompletely elucidated. On the other hand, *in vivo* efficacy, pharmacokinetic profiles, and long-term safety data of these agents remain severely lacking. Therefore, multi-dimensional translational research strategies are required to drive breakthroughs in future studies. First, structural biology techniques should be employed to identify the precise molecular targets of lead metabolites. Second, *in vivo* validation should be conducted in humanized mouse models of chronic HIV infection to systematically evaluate the agents’ inhibitory effects on viral load, regulatory roles in viral reservoirs, and tissue tropism. Meanwhile, structure-activity relationship (SAR) studies should be conducted to optimize metabolite structures, enhancing antiviral activity, metabolic stability, and oral bioavailability while reducing cytotoxicity. Through systematic exploration of the medicinal potential of *Kadsura* plants, it is expected to supplement existing antiretroviral therapy (ART) with novel mechanism-oriented anti-HIV lead metabolites, advancing the clinical translation of natural-source anti-HIV agents.

### 3.7 Antiplatelet aggregation effects

Studies have demonstrated the antiplatelet aggregation potential of *Kadsura* species. Specifically, seco-coccinic acid I, isovaleroylbinankadsurin A, and acetyl-binankadsurin A from *K. coccinea,* as well as heteroclitin D, (+)-anwulignan, meso-dihydroguaiaretic acid, and acid-kadsulignan L from *K. heteroclita* and *K. angustifolia*, all demonstrate significant antiplatelet aggregation activity (Association and Prevention, 2022; Su et al., 2017; Li et al., 1999a; Chen et al., 1998). Notably, among these metabolites, heteroclitin D effectively inhibited vascular constriction responses induced by high-potassium depolarization, calcium chloride, and norepinephrine (NA), and exerted a more pronounced inhibitory effect on platelet aggregation (Li et al., 1999a). Additionally, meso-dihydroguaiaretic acid and acid-kadsulignan L demonstrated antagonistic activity against platelet-activating factor (PAF) (Chen et al., 1998).

Platelet aggregation plays a pivotal role in activating blood circulation and eliminating blood stasis through the action of *Kadsura* plants (Jiang et al., 2005). This discovery not only explains the effectiveness of *Kadsura* genus plants in traditional Chinese medicine for treating various cardiovascular diseases but also opens up new avenues for modern medicine. However, the research on its mechanisms is not sufficiently in-depth; no studies have been conducted on toxicity or long-term effects, and individual differences have not been adequately considered. Future research should focus on elucidating mechanisms, optimizing doses, and conducting long-term toxicity and individual-difference studies to enhance the reliability of research findings and their clinical application.

### 3.8 Anti-leukemia effects

Current research has identified four *Kadsura* species with anti-leukemic activity: *K. coccinea* (Wang et al., 2008), *K. heteroclita* (Wang et al., 2006), *K. longipedunculata* (Liu and Huang, 1991; Liu and Pan, 1991), and *K. ananosma* (Chen et al., 2006; Yang et al., 2010). However, significant interspecific differences have been observed in their bioactive metabolite profiles and anti-leukemic efficacy. For instance, seco-coccinic acids A-C and seco-coccinic acid E isolated from *K. coccinea* significantly inhibit the proliferation of HL-60 human promyelocytic leukemia cells, with IC_50_ ranging from 6.8 to 42.1 µmol/L (Wang et al., 2008). In contrast, *K. longipedunculata* produces changanic acid, schisanlactone E, and schisanlactone F, which exhibit inhibitory effects on murine P-388 leukemia cells with IC_50_ values of 10 µg/mL, 1 µg/mL, and 5 µg/mL, respectively (Liu and Huang, 1991; Liu and Pan, 1991). Notably, while *K. heteroclita* and *K. ananosma* have been reported to possess anti-leukemic activity, their specific bioactive metabolites and underlying mechanisms remain largely uncharacterized, leaving critical gaps in our understanding of their therapeutic potential.

### 3.9 Neuroprotection effects

Plants of the genus *Kadsura* also exhibit notable neuroprotective potential. For instance, extracts from *K. heteroclita* significantly promote the growth and development of hippocampal neurons (Xiao et al., 2002). Ananolignans F and L isolated from *K. ananosma* exhibit potent neuroprotective effects (Yang et al., 2011). Additionally, specific metabolites from *K. japonica* and *Kadsura polysperma* Y.C.Yang also exert protective effects against neuronal injury (Dong et al., 2012; Liu et al., 2023).

Existing research lacks systematic identification of the active metabolites responsible for their neuroprotective effects, as well as studies on the underlying mechanisms. To advance the development and use of botanical drugs, future efforts should integrate modern research methodologies, such as multi-omics and single-cell technologies, to further explore their pharmacological value. This will facilitate the translation from basic research to clinical applications, enabling a shift from single-metabolite mechanism elucidation to multi-target system intervention. Such progress will provide novel botanical drug options for the treatment of neurodegenerative diseases.

### 3.10 Others

Beyond their shared pharmacological properties, individual *Kadsura* species exhibit distinct therapeutic potentials. For instance, the fruits of *K. coccinea* have the potential to prevent hyperlipidemia. The bitter taste-removing oils and polysaccharides in its fruits exert lipid-lowering effects. The underlying mechanism may involve enhancing the activity of superoxide dismutase, glutathione peroxidase, and catalase while reducing the levels of malondialdehyde, total cholesterol (TC), triglycerides (TG), and low-density lipoprotein cholesterol (LDL-C) (Zhang et al., 2022; Long et al., 2022). Additionally, its fruit extracts have preventive effects against bacterial diarrhea and effectively mitigate senna-induced diarrhea (Ling, 2021). Furthermore, the alcohol extract inhibits multiple venom enzymes from *K. coccinea* (Huang et al., 2020). For *K. heteroclita*, the leaf essential oil (EO) and its major chemical metabolites (δ-Cadinene, Calarene, and δ-4-Carene) exhibit varying degrees of cytotoxicity toward larvae of three mosquito vector species (Ling, 2021). These findings provide a basis for developing new, safer natural larvicides to combat diseases such as malaria and dengue fever. Meanwhile, the metabolites anwuweizonic acid, coccinic acid, and manwuweizic acid, isolated from the stems of *K. angustifolia*, exert significant antifertility effects (Chen et al., 2001; Chen Y. et al. 2002).

**FIGURE 2:**
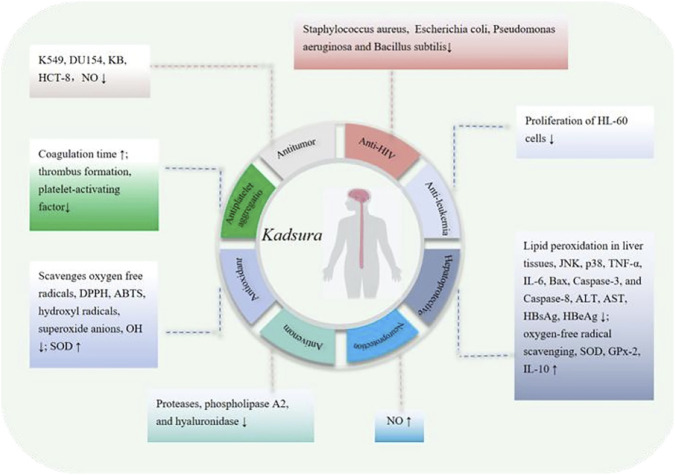
Main pharmacological effects of the *Kadsura* gene and their corresponding effect mechanisms.

In summary, plants of the genus *Kadsura* not only exhibit common pharmacological effects such as hepatoprotective (Qu et al., 2003), antibacterial (Feng et al., 2011), antioxidant (Ai and Li, 2005), antitumor (Jin, 2022), and anti-HIV(Xu, 2006) activities but also demonstrate diverse pharmacological properties, including lipid-regulating (Zhang et al., 2022), antidiarrheal (Ling, 2021), antifertility (Chen et al., 2001), anticomplementary (Huang, 2010), and insecticidal (Govindarajan et al., 2016) effects. These findings highlight the potential therapeutic value of various metabolites in *Kadsura* plants, indicating broad prospects for their development and utilization. Furthermore, these research outcomes provide a robust theoretical foundation for future drug development based on the *Kadsura* species. A recapitulative summary is presented in Table 2 and Figure 2.

**TABLE 2:** Pharmacological activities of *Kadsura* gene (“↓”, decrease; “↑”, increase).

Bioactivity	Metabolites/Extracts	Types	Testing subjects	Doses	Effects/Mechanisms	Control group	Refs
Hepatoprotective effects	Ethanol extract	*In vitro*	HepG2 cells	100 μM	TC levels ↓	FFA-induced group/DMEM Medium Supplemented with 10% FBS	Wu et al. (2024)
	Aqueous extract	*In vivo*	Clean male and female mice	2.5, 5.0 g/kg	ALT, AST↓ SOD↑	Colchicine/Normal Saline	Li et al. (2010)
	Ethanol extract	*In vivo*	SPF ICR mice	100, 200, 400 mg/kg	Serum alanine aminotransferase, aspartate aminotransferase levels, apoptosis, oxidative stress, p-Nrf2, Keap1, Cleaved caspase 3 ↓ glutathione, histopathological damage, Bcl-2/BAX ratio, HO-1, and Nrf2 expression↑	Bicyclol/Normal Saline	Wang et al. (2025)
	Aqueous extract	*In vivo*	KM male and female mice	20.75, 41.50, 83.00 g/kg	ALT, AST↓	Administer 200 mg/kg of bifendate/Normal Saline	Liu et al. (2015)
	Ethanol extract	*In vivo*	SPF SD male mice	0.42, 0.84, 1.68 g/kg	TGF-β1, TNF-α↓ IFN-γ↑	Administer Fufang Biejia Ruangan pills/Normal Saline	Liu et al. (2018c)
	Ethanol extract	*In vivo*	SPF SD male mice	1.5, 3.0, 6.0 g/kg	FFA, MDA↓ SOD↑	Administer simvastatin of 4 mg/kg/Normal Saline	Wang et al. (2015)
	Ethanol extract	*In vivo*	Clean male and female mice	3.0, 5.0 g/kg	ALT, AST, MDA↓ serum, SOD↑	Administer 25 mg/kg of bifendate/Normal Saline	Zhu (2007)
	Ethanol extract	*In vitro*	HSC-T6 cells	1.5, 3.0, 6.0 g/kg	HSC-T6 cell proliferation↓ miR-193↑	No HSC-T6 cells were added	Fan et al. (2022)
	Ethanol extract	*In vivo*	ICR male mice	100, 200, 400 mg/kg	SOD, GPx-2, Bax, Caspase-3, Caspase-8↓ TNF-α, IL-6, MDA, MPO, Bcl-2↑	Administer a mixture of 25% CCl4 and olive oil/Do not process	Yu et al. (2021)
	Ethanol extract	*In vivo*	SPF SD male mice	92, 184, 368 mg/kg	ALT, AST↓ GSH↑	Administer 2.1 mg/kg of bifendate/Normal Saline.	Shen et al. (2013)
	Ethanol extract	*In vivo*	ICR male mice	200, 400, 800 mg/kg	GPx-2, IL-10, SOD↓ TNF-α, IL-6, MDA, MPO↑	Administer 200 mg/kg of bifendate/Distilled water	Yu et al. (2017)
	Ethanol extract	*In vitro*	HepG-2 cells	10 μM	cell viability↑	AdministerBicyclol//DMEM Medium Supplemented with 10% fetal calf serum	Wang et al. (2022)
	Ethanol extract	*In vivo*	ICR male mice	100, 200, 400 mg/kg	Inhibited hepatocyte apoptosis; Bcl-2, Bax↑	Administer 30 mg/kg bifendate/Distilled Water	Yu et al. (2021)
	Aqueous extract	*In vivo*	Healthy mice	25.65, 51.3, 102.6 g/kg	ALT, AST↓	Administer 200 mg/kg bifendate/Distilled Water	Liu et al. (2016)
Antioxidant effects	Ethanol extract	*In vitro*	DPPH,·OH and O2-	0.025, 0.05, 0.075, 0.1, 0.2, 0.4, 0.6, 0.8 mg/m L	DPPH,·OH and O2-↓	Vitamin C/Blank solution	Li et al. (2020)
	Kadsuralignan F	*In vitro*	Melan-A cells	5.94, 11.87, 23.74 μM	The melanin synthesis↓	DPBS/kojic acid (1%)	Goh et al. (2013)
	Ethanol extract	*In vitro*	phagocytic cells	2.5, 5, 10, 20, 40 µM	IC_50_ value of 25.56 µM	Vitamin E/ethanol	Liu et al. (2018e)
	Aqueous extract	*In vitro*	DPPH, ABTS	1.0 mg/mL	DPPH, ABTS↓	Vitamin C/Distilled Water	Tang (2020)
	Ethanol extract	*In vitro*	DPPH	125, 250, 500, 1,000 mg/mL	DPPH↓	Vitamin C/ethanol	Yan et al. (2013)
	Aqueous extract	*In vitro*	DPPH	0.2, 0.4, 0.6, 0.8, 1.0, 1.2 mg/mL	DPPH↓	L-ascorbic acid/Distilled Water	Ye et al. (2023)
Anti-inflammatory and analgesic effects	Ethanol extract	*In vitro*	RAW264.7 cells	Not given	IC_50_ values of 8.15; IL-6↓	Methotrexate	Yang et al. (2021)
	Ethanol extract	*In vivo*	SPF ICR mice	200, 400, 800 mg/kg	Inhibiting RA-FLS cell apoptosis and the NF-κB pathway	Not given	Yang et al. (2022)
	Aqueous extract	*In vivo*	Clean SD mice	0.15, 0.3, 0.6 g/mL	TNF-α, IL-1β↓	Dexamethasone (0.5 g/lL)/Normal Saline	Li et al. (2014)
	Ethanol extract	*In vivo*	SPF SD mice	200, 400, 800 mg/kg	IL-1, IL-2, IL-6, IL-7, TNF-α↓α	Methotrexate (1 mg/kg)/Sodium carboxymethyl cellulose solution (0.3%)	Lu (2021)
	Ethanol extract	*In vitro*	RAW264.7 cells	1, 3, 10, 30, 50, 100 µg/mL	IC_50_ value of 34.07 µM	Lipopolysaccharide (100 ng/mL)/DMSO(1%)	Sun et al. (2022)
	Ethanol extract	*In vitro*	RAW264.7 cells	20, 50, 100 µg/mL	IL-6, IL-12, TNF-α, IL-1β↓	Not given	Li (2023)
	Ethanol extract	*In vivo*	SPF ICR mice	50, 100, 200 mg/kg	TNF-α,IL-1β,IL-6 ↓	Administer 200 mg/kg of bifendate/Distilled Water	Yu (2017)
	Ethanol extract	*In vivo*	SPF male mice	1.0, 2.0, 4.0 mg/kg	Promoting apoptosis of mature osteoclasts, inhibiting osteoclast differentiation, and reducing bone resorption; RANKL, RANK, NFATc1, MMP-9, CTSK, TRAP↓	Sodium carboxymethyl cellulose solution (0.3%)	Zheng et al. (2024)
	Ethyl acetate extract	*In vivo*	SPF mice	4.2, 8.4, 16.8 mg/mL	IL-1β, IL-6, TNF-α↓	Dexamethasone (0.15 mL/kg)/Normal Saline	Tan et al. (2017)
Antiplatelet aggregation effects	Aqueous extract	*In vivo*	SPF mice	7.5, 15 g/kg	Prolonged coagulation time	Warfarin sodium (0.52 mg/kg)/Distilled Water	Su et al. (2017)
	Ethanol extract	*In vivo*	New Zealand White Rabbits	0.05, 0.5, 1, 5 mg/L	ADP and PAF-induced platelet aggregation↓	Aspirin (10 mg/L)/Absolute ethyl alcohol	Jiang et al. (2005)
Anti-leukemia effects	Ethanol extract	*In vitro*	HL-60 cells	Not specified	GI_50_ values of 6.8–42.1 µM	5-Fluorouracil (50%)/DMSO(0.1%)	Wang et al. (2008)
Neuroprotection effects	Methanolic extract	*In vitro*	BV2 murine microglial cells	1–50 μg/mL	Regulation of iNOS expression and NF-κB nuclear translocation	NF-κB inhibitor	Liu et al. (2023)
Hypolipidaemic effects	Heilaohu seed oil	*In vivo*	SPF male mice	150, 300 μL/kg	TC, TG, LDL-C↓ HDL-C, SOD, T-AOC↑	Feed a high-fat diet/Distilled Water	Zhang et al. (2022)
	Ethanol extract	*In vivo*	Male C57BL/6N mice	300 mg/kg	TC, TG, LDL-C↓ HDL-C↑	Fed a high-fat diet	Long et al. (2022)

## 4 Molecular pharmacognosy

### 4.1 Chloroplast genome and phylogeny

**FIGURE 3:**
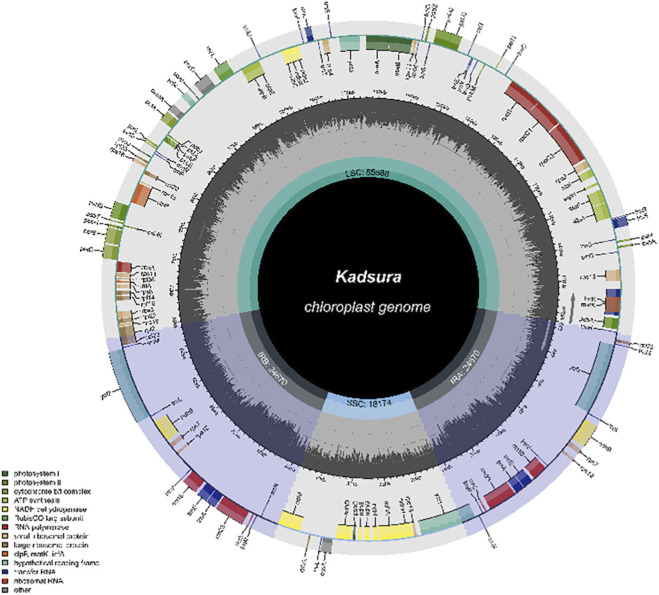
The chloroplast genome of *Kadsura* species.

Currently, there has been relatively extensive research on the chloroplast genome of *Kadsura* species (Figure 3). In 2017, Guo (2017) conducted the first study on the chloroplast genomes of multiple *Kadsura* species, revealing that *K. heteroclita*, *K. longipedunculata*, *K. japonica*, *K. interior*, and *K. oblongifolia* share close phylogenetic relationships. Subsequently, numerous studies on the chloroplast genome of *Kadsura* have been published, with detailed information summarized in Table 3. Table 2 shows that the chloroplast genome lengths of *Kadsura* species range from 145,399 bp to 153,362 bp, with the longest being *K. heteroclita* and the shortest being *K. coccinea*. The lengths of the LSC, IR, and SSC regions range from 85,243 bp to 94,757 bp, 16,431 bp to 24,673 bp, and 17,945 bp to 18,253 bp, respectively. The total number of genes ranges from 113 to 129, with protein-coding genes, rRNA genes, and tRNA genes ranging from 79 to 85, 4 to 8, and 30 to 37, respectively.

**TABLE 3:** Summary of chloroplast genome information of the *Kadsura* genus.

Species names	Number	Size (bp)	LSC length (bp)	IR length (bp)	SSC length (bp)	GC content (%)	Total genes	Protein-coding genes	rRNA genes	tRNA genes	References
*K. coccinea*	HLH-1A	145617	94481	16552	18032	39.7	125	82	8	35	Zhai et al. (2024)
	HLH-2A	145617	94481	16552	18032	39.7	125	82	8	35	Zhai et al. (2024)
	MN480469	145608	94457	16552	18047	39.7	125	82	8	35	Zhai et al. (2024)
	MT934443	145413	94511	16431	18040	39.7	125	82	8	35	Zhai et al. (2024)
	BN002	145875	94725	16552	18046	39.7	113	79	4	30	Guo (2017)
	2015091601	145412	94301	16536	18039	39.7	113	79	4	30	Guo (2017)
		145413	94301	16536	18040	39.7	113	79	4	30	Li and Zheng (2018)
		145413	94511	16431	18040	39.7	126	82	8	35	Qin et al. (2021)
	116600	145399	94287	16535	18039	39.7	124	82	8	34	Zhang et al. (2020)
	MN480469	145608	94457	16552	18047	38.6	126	83	7	35	Yang et al. (2019)
		145399	94287	16535	18039	39.7	124	82	8	34	Wang (2019)
*K. longipedunculata*	2015090802	153029	85515	24670	18174	39.6	113	79	4	30	Guo (2017)
	MW801021	153106	85593	24670	18173	39.6	129	84	8	37	Zhai et al. (2024)
*K. ananosma*	MN 823697/NC_0 57265	145903	94757	16552	18042	39.7	125	82	8	35	Zhai et al. (2024), Liu et al. (2020)
*K. heteroclita*	NC_050348	153201	85774	24656	18115	39.6	129	84	8	37	Zhai et al. (2024)
	MW801021	153106	85593	24670	18173	39.6	129	84	8	37	Zhai et al. (2024)
	2015092104	153127	85534	24670	18253	39.6	113	79	4	30	Guo (2017)
	2015082902	153096	85577	24670	18179	39.6	113	79	4	30	Guo (2017)
	2015090204B	153362	85916	24642	18162	39.6	113	79	4	30	Guo (2017)
	BN005	153108	85829	24667	17945	39.6	113	79	4	30	Guo (2017)
	MN823698	153289	85774	24657	18201	39.6	129	84	8	37	Wang et al. (2020)
*K. interior*	2015121202	152810	85285	24663	18199	39.6	113	79	4	30	Guo (2017)
	MN698966	153201	85774	24673	18077	39.6	129	85	8	37	Fu et al. (2020)
*K. oblongifolia*	HN2015110801	152753	85243	24670	18170	39.6	113	79	4	30	Guo (2017)
*K. japonica*	TBY2015120104	153027	85514	24670	18173	39.7	113	79	4	30	Guo (2017)

In addition, researchers conducted the phylogenetic analysis of the *Kadsura* plant and its closely related species using chloroplast genomes. For example, Wang et al. (2003) were the first to perform a sequence analysis of the chloroplast rbcL gene in Schisandraceae plants. Their study suggested a close relationship between the genera *Schisandra* Michx. and *Kadsura*, with interconnections and overlaps, leading to the hypothesis that the two genera may share a common ancestor. Li (2019) categorized the boundaries of the IR region in Schisandraceae into the S type and the L type, and species of the genus *Kadsura* include both types. Specifically, *K. coccinea* has an S-type IR region, whereas the other *Kadsura* species have an L-type IR region. Moreover, phylogenetic analysis shows that the genera *Kadsura* and *Schisandra* are non-monophyletic. It is also recommended that *Schisandra plena* A.C.Sm. and *Schisandra propinqua* (Wall.) Baill. be transferred to the genus *Kadsura*. From these results, it can be inferred that the genera *Schisandra* and *Kadsura* are non-monophyletic and probably share a common origin.

As can be seen from the above, researchers have completed the sequencing assembly and structural analysis of multiple chloroplast genomes of *Kadsura* species, and have used these data to conduct partial phylogenetic evolution analysis of *Kadsura* and its close relatives, providing a good data foundation for the later development of DNA barcode labeling and molecular marker-assisted breeding of this genus.

### 4.2 Transcriptional regulatory networks and functional genes

Research on secondary metabolic pathways and functional genes in *Kadsura* species is burgeoning. For example, Liang et al. (2022) conducted targeted metabolomic analysis combined with transcriptomic sequencing of the roots, stems, and leaves of *K. coccinea*. The metabolomic analysis identified 51 lignans, while transcriptomic sequencing revealed 137 unique genes across 13 categories of lignan biosynthesis. It was found that the CCoAOMT, C3H, and SIDR gene families were primarily expressed in the roots and stems. Further integrated analysis of metabolomics and transcriptomics identified 11 key enzyme gene families closely associated with lignan synthesis, including HCT, DIR, COMT, CAD, SIDR, and PLR. These findings demonstrate that *K. coccinea* roots, stems, and leaves contain numerous lignans and their associated synthesis enzyme genes, but the content and expression levels of these lignans and enzymes vary significantly among plant parts. Similarly, Fu et al. (2024) used transcriptomic sequencing to analyze root, stem, and leaf samples of *K. coccinea* and identified CYP genes, and conducted phylogenetic analyses of these genes. They were distributed across 8 clades within 38 families.

The roots exhibited specific expression patterns for CYP genes, and sequence alignment identified 22 homologous single genes among these CYPs. Of these, six homologous genes of CYP719As and one gene of CYP81Qs were highly expressed in the roots. Wang et al. (2019) conducted transcriptome sequencing of the roots, stems, and leaves of *K. coccinea*, successfully cloning KcSQS and performing bioinformatics analysis. This provides a basis for further research into the biosynthesis of triterpenoids in *K. coccinea*. In addition, Li et al. (2022) studied the leaves of *K. ananosma* and obtained transcriptomic data, laying the foundation for exploring the biosynthetic pathways and functional genes of active metabolites in *K. ananosma*.

In conclusion, research on the transcriptional regulatory network and the functional genes of *Kadsura* plants is still in its infancy. The main research focus is on *K. coccinea*. People have only conducted preliminary screening and expression profiling studies on some key regulatory genes at the transcriptomic and metabolomic levels, and have not carried out further functional verification. However, relevant research on other species remains a blank.

### 4.3 Comparative genomic research

To date, there have been very few studies on the genomes of *Kadsura* plants (Xu et al., 2021). Estimated the genome sizes of four *Kadsura* species (*K. interior*, *K. heteroclita*, *K. longipedunculata*, and *K. coccinea*) using FCM. The comparison revealed minimal differences in genome size among *K. interior*, *K. heteroclita*, and *K. longipedunculata*, whereas *K. coccinea* showed a markedly distinct genome size. This indicates that genome size alone is insufficient for fully distinguishing these species. Dong (2022) studied a total of 107 individuals of *K. interior*, *K. heteroclita*, *K. longipedunculata*, *K. oblongifolia*, and *K. coccinea* to examine genetic diversity, phylogeny, and ecology using single-nucleotide polymorphisms (SNPs) generated via restriction site-associated DNA sequencing (RAD-seq). The results showed moderate differentiation among *K. heteroclita*, *K. longipedunculata*, and *K. oblongifolia*, and a high degree of genetic differentiation between *K. interior* and these species. The phylogenetic tree indicated that each species was monophyletic. Furthermore, the results of population genetic structure showed that there was admixture and gene flow among *K. heteroclita*, *K. longipedunculata*, and *K. oblongifolia*. Moreover, due to morphological similarities, “Flora of China” treated *K. interior* and *K. heteroclita* as conspecific. However, based on the above research, Dong (2022) proposed that *K. interior* should not be considered a synonym of *K. heteroclita*.

In addition, Lee et al. (2023) assembled the genomes of *Schisandra repanda* (Siebold and Zucc.) Radlk. and *K. japonica* using a combination of Nanopore and Illumina sequencing technologies. They employed these genomes for taxonomic studies and developed two InDel markers to differentiate *S. repanda*, *K. japonica*, and *Schisandra chinensis* (Turcz.) Baill. This methodology offers a new avenue for exploring the evolutionary relationships within the Schisandraceae family.

In summary, to date, only a preliminary assessment of genome size and degenerate genome sequencing analysis have been conducted on some plants of the *Kadsura* genus, and the resulting data have been used for species identification and phylogenetic analysis of the genus. Whole-genome sequencing and further research on molecular regulatory mechanisms, transcriptional regulatory networks related to plant growth and development, or secondary metabolites have not yet been carried out.

## 5 Conclusion and future prospects

From the above review, we can see that people consume the fruits of many *Kadsura* species, which have definite medicinal value, such as *K. coccinea*, *K. heteroclita*, and *K. oblongifolia*. Among them, *K. coccinea* is currently the most widely used, extensively studied, and largest cultivated species. In addition, most plants of the *Kadsura* genus have the effects of promoting qi circulation and relieving pain, activating blood circulation and removing blood stasis, dispelling wind and dampness. Meanwhile, some species also have unique medicinal properties, such as *K. interior*, *K. japonica*, *K. oblongifolia,* and *K. ananosma*—their unique chemical metabolites determine the distinctive medicinal value of these species. Previously, people have conducted relatively detailed research on the chemical metabolites of the genus *Kadsura*. However, to date, researchers have not conducted corresponding pharmacological activity verification and active metabolite analysis for the above-mentioned species with unique traditional medicinal value. Perhaps through these studies, novel active metabolites can be discovered, and data supporting the development of new medicines can be provided.

In terms of pharmacological activity, plants of the genus *Kadsura* not only exhibit common effects such as hepatoprotective, antibacterial, antioxidant, antitumor, and anti-HIV activities but also demonstrate diverse pharmacological properties, including lipid-regulating, antidiarrheal, antifertility, and insecticidal effects, among others. These findings highlight the potential therapeutic value of various metabolites in *Kadsura* plants, indicating broad prospects for their development and utilization. Furthermore, these research outcomes provide a robust theoretical foundation for future drug development based on the *Kadsura* species. A recapitulative summary is presented in Table 2 and Figure 2. It should be noted that the current research results indicate that plants of the *Kadsura* genus exhibit unique attributes in both traditional folk disease applications and modern metabolite composition and pharmacological activity studies. However, systematic comparative studies of different *Kadsura* species have not been conducted to elucidate the scientific significance of the unique attributes of individual species. It can be inferred that implementing such research will significantly advance the discovery of novel metabolites and the advancement of new drug development.

Furthermore, as can be seen from Tables 1, 3, many plants of the *Kadsura* genus have excellent medicinal value. However, the species that have been extensively studied in modern pharmacology are limited to a few, such as *K. cocinea*, *K. heteroclita*, and *K. longipedunculata*. As mentioned above, some *Kadsura* species have unique medicinal properties, such as *K. interior*, *K. japonica*, *K. oblongifolia*, and *K. ananosma*; however, pharmacological research on these species is relatively scarce. Therefore, we suggest that researchers shift their focus to these species to discover more valuable pharmacological activities and development potential.

Regarding propagation techniques, the most commonly used method for *Kadsura* plants is cutting propagation, and seed propagation is rarely adopted, which might be related to the fact that all *Kadsura* plants are vines (with longer growth cycles), and cutting propagation can shorten the growth cycle. Currently, research on seed propagation techniques has been reported only for *K. coccinea.* Research has shown that various methods can effectively break *K. coccinea* seed dormancy, promoting germination. However, whether these methods can also be applied to other *Kadsura* species requires further experimental verification. Research on tissue culture of the genus *K. coccinea* has just begun, and only partial work has been carried out on *K. coccinea*. Tissue culture is not only an effective method for enhancing breeding efficiency but also a fundamental tool for genetic transformation and functional gene research in plants. Therefore, it is imperative to conduct tissue culture for plants of the genus *Kadsura*.

In the field of molecular pharmacognosy, researchers have completed the sequencing assembly and structural analysis of multiple chloroplast genomes of *Kadsura* species, and have used these data to conduct partial phylogenetic evolution analysis of *Kadsura* and its close relatives, providing a good data foundation for the later development of DNA barcode labeling and molecular marker-assisted breeding of this genus. Meanwhile, research on the transcriptional regulatory network and the functional genes of *Kadsura* plants is still in its infancy; the main focus is on *K. coccinea*, and only preliminary screening and expression profiling studies have been conducted on some key regulatory genes at the transcriptome and metabolome levels. Furthermore, whole-genome sequencing and further research on molecular regulatory mechanisms underlying plant growth and development or secondary metabolite production have not yet been conducted. It is now extremely urgent to carry out whole-genome sequencing for *Kadsura* species with significant developmental value, as well as to verify functional genes and to develop molecular marker-assisted breeding based on this.

In conclusion, we propose three priority research directions: First, systematically validate species-specific pharmacological activities and characterize their bioactive metabolites; second, intensify research on tissue culture propagation techniques; third, accelerate whole-genome sequencing of priority *Kadsura* species, alongside gene screening, functional validation, and molecular regulatory network analysis, to lay a solid foundation for biosynthetic pathway elucidation and molecular marker-assisted breeding of elite cultivars.

## Glossary

6-BA: 6-benzylaminopurine
3-IBA: 3-Indole butyric acid
ADP: adenosine diphosphate
AIA: antigen-induced arthritis
AMPK: AMP-activated protein kinase
BMI: body mass index
Bax: Bcl2-Associated X
Bcl-2: β-cell lymphoma-2
CYP: cytochrome P450
CTSK: cathepsin K
COX-1: cyclooxygenase-1
CNS: central nervous system
COX-2: cyclooxygenase-2
NO: nitric oxide
EO: essential oil
FCM: flow cytometry
GABA A: gamma-aminobutyric acid type A
GA: gibberellin
GPx-2: glutathione peroxidase-2
iNOS: inducible nitric oxide synthase
IR: inverted repeat
IAA: Indole acetic acid
LSC: large single-copy
ALT: Alanine aminotransferase
AST: Aspartate aminotransferase
TGF-β1: Transforming growth factor beta 1
FFA: Free fatty acids
RANKL: Receptor activator of NF-kB ligand
MMP-9: Matrix metalloproteinase-9
TRAP: Targeted Recombination in Active Populations
T-AOC: Total antioxidant capacity
MDA: malondialdehyde
MPO: myeloperoxidase
NF-κB: nuclear factor κB
RAD-seq: reduced-representation genome sequencing
NA: norepinephrine
**O** ^**2-**^: superoxide anions
PAF: platelet-activating factor
ROS: reactive oxygen species
DPPH: 2,2-diphenyl-1-picrylhydrazyl
rRNA: ribosomal RNA
SOD: superoxide dismutase
SNP: single nucleotide polymorphism
SA: salicylic acid
TCM: traditional Chinese medicine
tRNA: transport RNA
TRAP: tartrate-resistant acid phosphatase
TC: total cholesterol
TG: triglycerides
**IC**_**50**_: 50% inhibitory concentration
IL-1β: interleukin-1β
KcSQS: squalene synthase gene
HDL-C: high-density lipoprotein cholesterol
LDL-C: low-density lipoprotein cholesterol
Nrf-2: Nuclear factor erythroid 2-related factor 2
IFN-γ: Interferon gamma
GSH: Glutathione
NFAT: Nuclear factor of activated T cells
**GI**_**50**_: 50% Growth Inhibition Concentration
DMSO: Dimethyl Sulphoxide
